# Diodicity of MicroTesla Valves Under Various Re Numbers

**DOI:** 10.3390/mi16121329

**Published:** 2025-11-26

**Authors:** Christos Liosis, Alexandros Papadatos, Dimitrios-Nikolaos Pagonis, Sofia Peppa, Ioannis Sarris

**Affiliations:** 1 Department of Mechanical Engineering, University of West Attica, 12241 Athens, Greece; liosischristos@uniwa.gr; 2 Department of Biomedical Engineering, University of West Attica, 12243 Athens, Greece; 3 Department of Naval Architecture, University of West Attica, 12243 Athens, Greece; na20393067@uniwa.gr (A.P.); d.n.pagonis@uniwa.gr (D.-N.P.); speppa@uniwa.gr (S.P.)

**Keywords:** Tesla valve, OpenFoam, diodicity, Reynolds number, microfluidics

## Abstract

Although the Tesla valve is a well-known technology spanning almost 100 years, its wide range of potential applications in modern engineering problems has made it particularly attractive to researchers in the last few years. The major factor that characterizes the Tesla’s valve effectiveness is the diodicity (*D*), which is practically defined as the ratio of the pressure difference in reverse to forward flow $D=\frac{\Delta P_{rev}}{\Delta P_{for}}$. Under this framework, a geometry of multi-staged Tesla valves was selected to investigate the correlation between the Reynolds (Re) number and diodicity. Initial simulations were performed for N=2, N=6 and N=10 multi-staged micro Tesla valves using the OpenFoam platform, with Reynolds numbers of Re 50–450. Here, the maximum diodicity values obtained were D=1.43, D=2.76 and D=3.58 for double-, six- and ten-staged micro Tesla valves under Re=450, respectively. Further simulations were performed for N=3 and N=5 under the same initial conditions in order to investigate the proportionality between N and D.

## 1. Introduction

The necessity of addressing problems extending from the microscale to macroscale has led to the exploration of past theories and practical implementations as sources of solutions to these physical problems. One promising concept is the Tesla valve, which operates passively, using only its geometry to create asymmetric flow resistance. When the fluid flows through the forward direction, the pressure drop is lower than in the reverse-direction flow, whereas, in the reverse flow, higher flow resistance arises. Based on these principles, the Tesla valve can be implemented in various applications across fields such as medical devices [1,2,3], chemical processing [4,5] and applied physics, including mixing, heat sinks and pumps [6,7,8]. Although a common characteristic of the above applications is that they involve microfluidic devices, the Tesla valve could find applicability in macroscale applications, such as in the marine [9] and health [10] fields. Moreover, based on flow control, the Tesla valve has also achieved significant results in various other fields [11,12,13,14].

The diodicity (D) is an indicator of whether the geometry has good performance, defined as the ratio of the pressure drop in reverse flow to that in forward flow [15]. Several studies have emphasized the optimization of the valve geometry [16,17] in order to achieve higher diodicity, while other studies have focused on the relationship between the Reynolds number (Re) and diodicity [18,19]. The main characteristics of the Tesla valve are the contact angle, the total length of the valve, the number of staged valves, the shape of the loops, and the inlet–outlet dimensions. All these characteristics can be optimized in order to maximize D.

The double Tesla valve (N=2) was investigated in our previous work as a micromixer [20,21], with higher performance compared to other micromixers and geometries. Since the geometry is functional as a micromixer for very low Re ≈ 2 but not optimized, the present work focuses on the relation between D and Re, focusing on Re up to 450 for the existing geometry. The primary objective of this work was to find a critical Re where, during the reverse flow, the velocity is higher within the loops and exceeds the main path. Additionally, this work aimed to study the valve performance and investigate the effects of additional valves for various Re under the same initial conditions on D.

## 2. Materials and Methods

**Figure 1:**
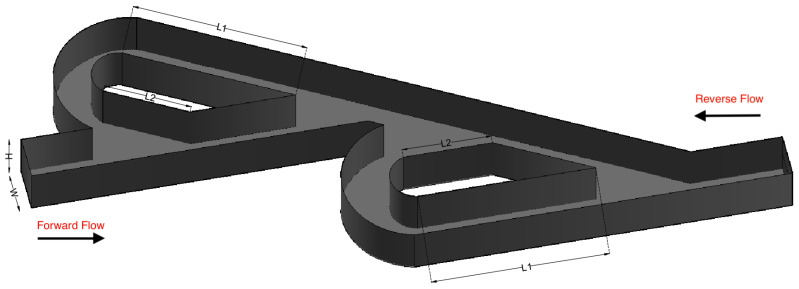
Double micro Tesla valve geometry.

**Figure 2:**
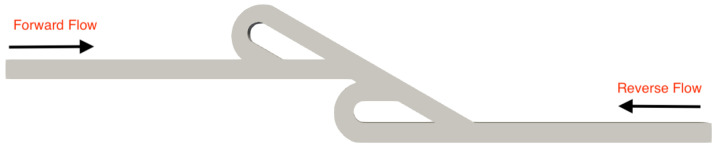
Extended double micro Tesla valve geometry.

**Figure 3:**
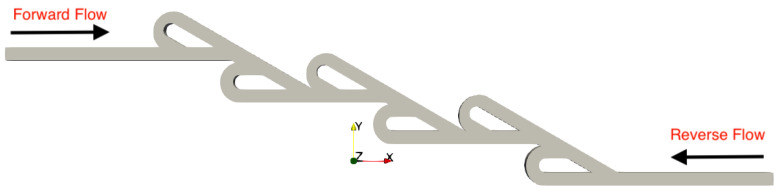
Six-staged micro Tesla valve geometry.

**Figure 4:**
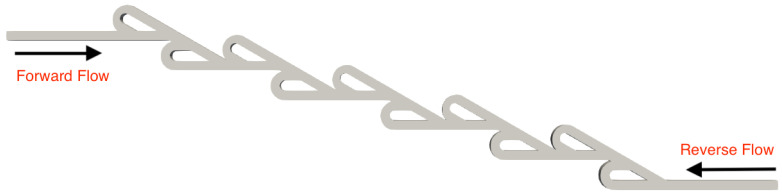
Ten-staged micro Tesla valve geometry.

A series of simulations was performed for both micro Tesla valve geometries under various Reynolds numbers (Re). The Tesla micromixer geometry uses two units of valves that are connected in series, with both the inlet and outlet of the micromixer being a squared cross-section of height and width $W=H=10^{-4}$ m, corresponding to the length of each side (a). The length ratio of $\frac{L_{1}}{L_{2}}=\frac{375\;\mu m}{187.5\;\mu m}=2$ was selected from an existing Tesla structure [22]. The geometry of the double Tesla (N=2) multi-staged valve design is identical to that in our previous work [21] and is illustrated in Figure 1. In the present simulations, both the inlet and the outlet were extended by 15 mm according to [18], as shown in Figure 2. Furthermore, the six-staged (N=6) and ten-staged (N=10) valves are based on the double Tesla, as shown in Figure 3 and Figure 4, respectively.

Since the inlet for a forward or reverse flow is a square, the Re can be calculated from [23]

(1) $$Re=\frac{u\cdot D_{H}}{\nu }=\frac{u\cdot \rho \cdot D_{H}}{\mu }=\frac{u\cdot \rho \cdot a}{\mu }$$

where, for the fluid (water), *u* (m/s) is the velocity, $\nu =10^{-6}$ m^2^/s is the kinematic viscosity, $\rho =10^{3}$ kg/m^3^ is the density, and $\mu =10^{-3}$kg/m·s is the dynamic viscosity. $D_{H}$ is the hydraulic diameter of the pipe and *a* represents the side length of the square cross-section. In both cases, the Re number depends on the fluid velocity, since all other parameters remain constant during the simulations. Thus, the above equation can be simplified as

(2) $$Re=10^{2}\left(s/m\right)\cdot u\left(m/s\right)$$

Another interesting parameter is the volumetric flow rate, which can be calculated from

**Figure 5:**
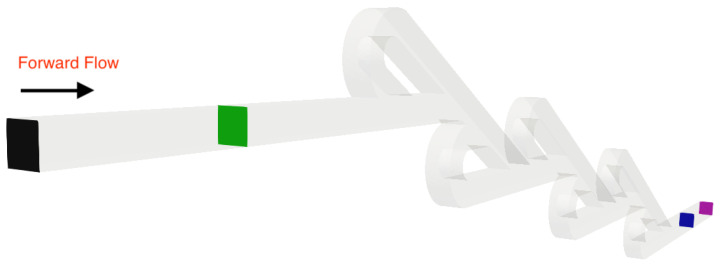
Patches where the measurements for average pressure were taken for the six-staged micro Tesla valve under forward flow.

**Figure 6:**
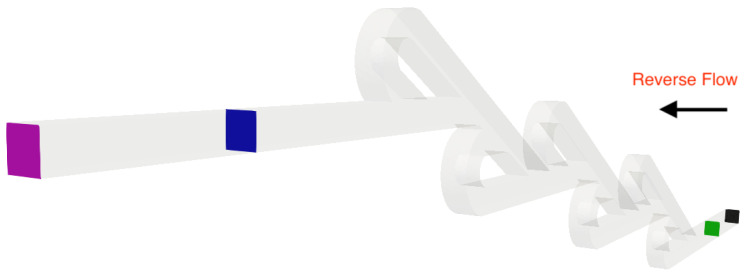
Patches where the measurements for average pressure were taken for the six-staged micro Tesla valve under reverse flow.

(3) Q=A·u

where *u* (m/s) is the velocity of the fluid and $A=10^{4}\;\mu m^{2}=10^{-8}\;m^{2}$ is the inlet surface area in this study. Under the microscale used, *Q* is expressed in μL/s. The diodicity (D) is a performance indicator for the Tesla valve and is defined as the ratio

(4) $$D=\frac{\Delta P_{rev}}{\Delta P_{for}}$$

where $\Delta P_{rev}$ is the pressure difference between $P_{inlet}-P_{outlet}$ for the reverse flow and $\Delta P_{for}$ is the pressure difference between $P_{inlet}-P_{outlet}$ for the forward flow. Additionally, the entrance and exit lengths were extended by 1.2 mm in order to resolve any potential issues at the outlet boundary condition for the reverse flow [18]. The inlet and the outlet slices for all scenarios are colored black and purple, respectively. Moreover, the average pressure was measured at four different locations (patches) for each simulation, as illustrated in Figure 5 and Figure 6. Thus, the diodicity was first evaluated using the patches at the inlet and the outlet (where the mesh validation was performed) and re-evaluated using intermediate patches (blue and green) that were located 0.6 mm from the inlet and outlet boundaries of the geometry, following the procedure in [18]. The same approach was applied for the double- and ten-staged micro Tesla valves.

Moreover, all geometries and computational meshes were created using the open-source software GMSH (version 4.13.1). The simulations were performed with OpenFoam (version 10), which is also a free software platform. In these simulations, the velocity and pressure fields were obtained by employing a suitable solver that corresponded to the problem requirements. The solver was based on the Semi-Implicit Method for Pressure-Linked Equations (SIMPLE). The governing equations were the continuity (5) and momentum (6) equations. The above equations were solved for the fluid phase and are based on the incompressible Navier–Stokes equations, where *p* and *u* are the pressure and velocity, respectively. The viscosity and density of the water are denoted by μ and ρ, respectively:

(5) ∇·u=0

(6) $$\rho \;\left(u\cdot \nabla \right)u=-\nabla p+\mu \nabla^{2}u$$

The use of computational fluid dynamics demands a mesh independence study, which was performed for all geometries considered. Since the evaluation of the diodicity required two separate simulations for each case (one for the forward and one for the reverse flow), this increased the computational cost. Therefore, the selection of the most suitable mesh was crucial in balancing accuracy and efficiency. To achieve this, two mesh independence studies were performed for the forward and reverse flows, followed by an estimation of the diodicity error. Specifically, the mesh independence analysis was carried out for the following initial conditions: inlet velocity equal to u=4.5 m/s, corresponding to Re=450, which represents the highest inlet velocity and consecutively the highest Re among the simulated cases. Experimental investigations on soft-walled microchannels [24] reported attainable Reynolds numbers in the range of 400–500, limited by the bonding strength and pressure constraints of the microfabricated devices. The pressure difference value ΔP used for mesh validation was measured at the inlet and outlet of the geometry for all selected meshes (coarse, medium and fine) for all valve configurations. Additionally, the growth rate of the elements was kept constant (≈2) for the different meshes, as presented in Table 1.

The Richardson extrapolation method and grid convergence index (CGI) were applied for the mesh validation. The indices 1, 2 and 3 correspond to coarse, medium and fine meshes, respectively. Regarding $CGI_{23}$, which is the convergence between the medium and fine meshes, it should be noted that $CGI_{23}$ had differences between the flows. More specifically, for the double Tesla, $CGI_{23}$ was 0.3% and 1% for the forward and reverse flow, respectively. This difference arose mostly from the phenomena that take place in reverse flow. The diodicity and ΔP (forward and reverse) relative errors were calculated and expressed as percentages, and they were our final criteria for mesh validation. Initially, $\Delta P_{er,forward}$ and $\Delta P_{er,reverse}$ were calculated, followed by $D_{er}$.

Using the above calculations, $D_{er}$, $\Delta P_{er,forward}$ and $\Delta P_{er,reverse}$ were lower for the medium mesh compared to the coarse one. The diodicity error was Der=1.70%, Der=0.18% and Der=1.54% for the double-, six-staged and ten-staged cases, respectively. Thus, the mesh that was used for all simulations was the medium mesh.

**Figure 7:**
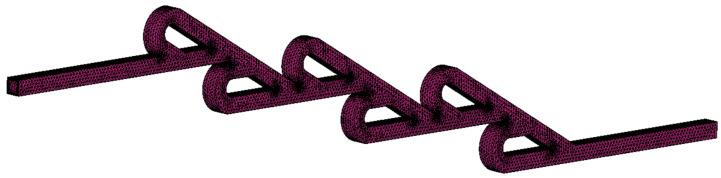
Mesh for six-staged micro Tesla.

**Figure 8:**
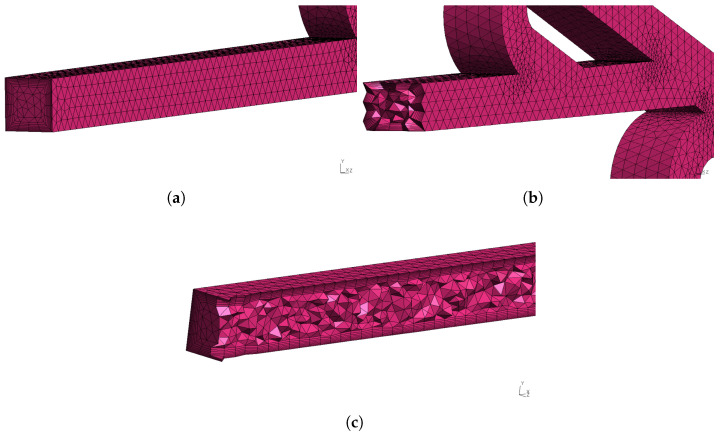
Six-staged micro Tesla mesh: (**a**) inlet/outlet magnified view, (**b**) cross-sectional slice, (**c**) vertical slice.

The mesh for the six-staged micro Tesla is presented in Figure 7. Additionally, the growth rate of the boundary layers was 1.18, and it consisted of 8 layers. More detailed views of the mesh and boundary layers are presented in Figure 8. Moreover, the meshes for the double- and ten-staged valves were created accordingly following the same mesh strategy. Although using different mesh resolutions for each Re could have reduced the computational cost, this approach requires a mesh independence study for each case, increasing the overall simulation workload. Therefore, under all simulations (different Re), the mesh was kept constant, since it had been validated for Re=450, which corresponded to the most demanding flow conditions.

The main simulation parameters, including the boundary conditions and physical properties of the water, are summarized in Table 2. Focusing on the inlet boundary condition and using Equations (2) and (3), the Re number and volumetric flow rate could be calculated, respectively. Based on these calculations, Table A1 was obtained, where the ranges of Re and Q are presented. In essence, Re varied from 50 to 450 in increments of 25, which indicated that the flow was laminar. The volumetric flow rate was used as an indicator for the potential applicability of the valve. The range of Q was from 5 to 45 with increments of 2.5 μL/s.

## 3. Results

**Figure 9:**
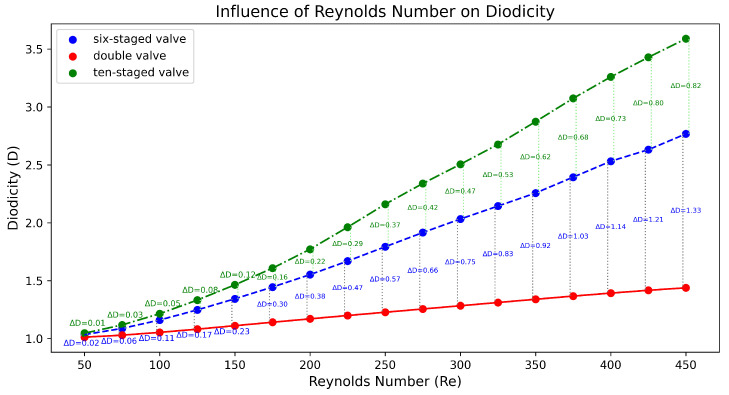
Diagram indicating how Re influences diodicity for all micro Tesla valve geometries.

**Figure 10:**
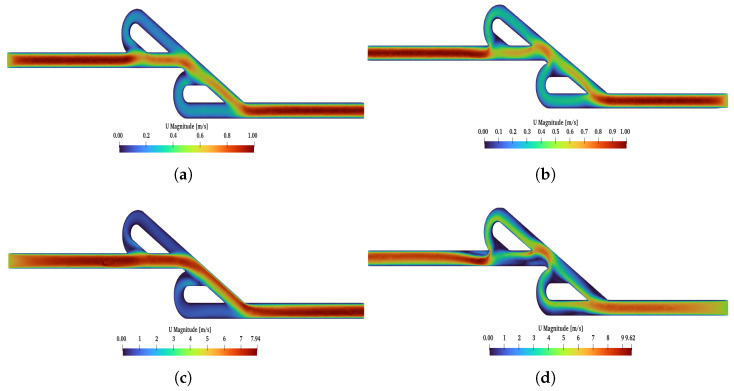
Velocity field of double Tesla under (**a**) Re = 50 and forward flow, (**b**) Re = 50 and reverse flow, (**c**) Re = 450 and forward flow, (**d**) Re = 450 and reverse flow.

The post-processing analysis was performed with Paraview (version 5.13.3), which is a free software program, while the diagrams were created in Python (version 3.9.6). The results of the double Tesla simulations (at inlet–outlet patches) are presented in Figure 9 with a red line. Measurements of the average pressure at the inlet and outlet were obtained for the black and purple patches (slices), respectively. As the Re increased, the diodicity of the double Tesla valve also increased. The maximum diodicity was achieved for Re=450 and was equal to D=1.43. Additionally, with decreasing Re, the diodicity will be decreased. The diodicity must always be ≥1, but it is very close to 1 for a low Re. In the present simulations, the minimum diodicity occurred under Re=50 and was found to be equal to 1.01. This arose from the fact that less resistance was generated, so the pressure drop in the reverse and forward flows became closer [23]. In Figure 10, the main differences between these two cases (Re=450, Re=50) are presented. When the reverse flow was applied, and for higher Re at the second loop, a higher velocity was observed, while, at the main path, the velocity decreased after the first loop. The maximum magnitude of the velocity occurred among the first and second loops. Additionally, for Re=50, the maximum velocity in the valve was similar for both flows, while, for Re=450, the maximum velocity was higher for the reverse than the forward flow. Finally, for forward flow, the velocity field had the same behavior for both Re numbers, and the magnitude of the velocity was higher at the main path, as expected.

**Figure 11:**
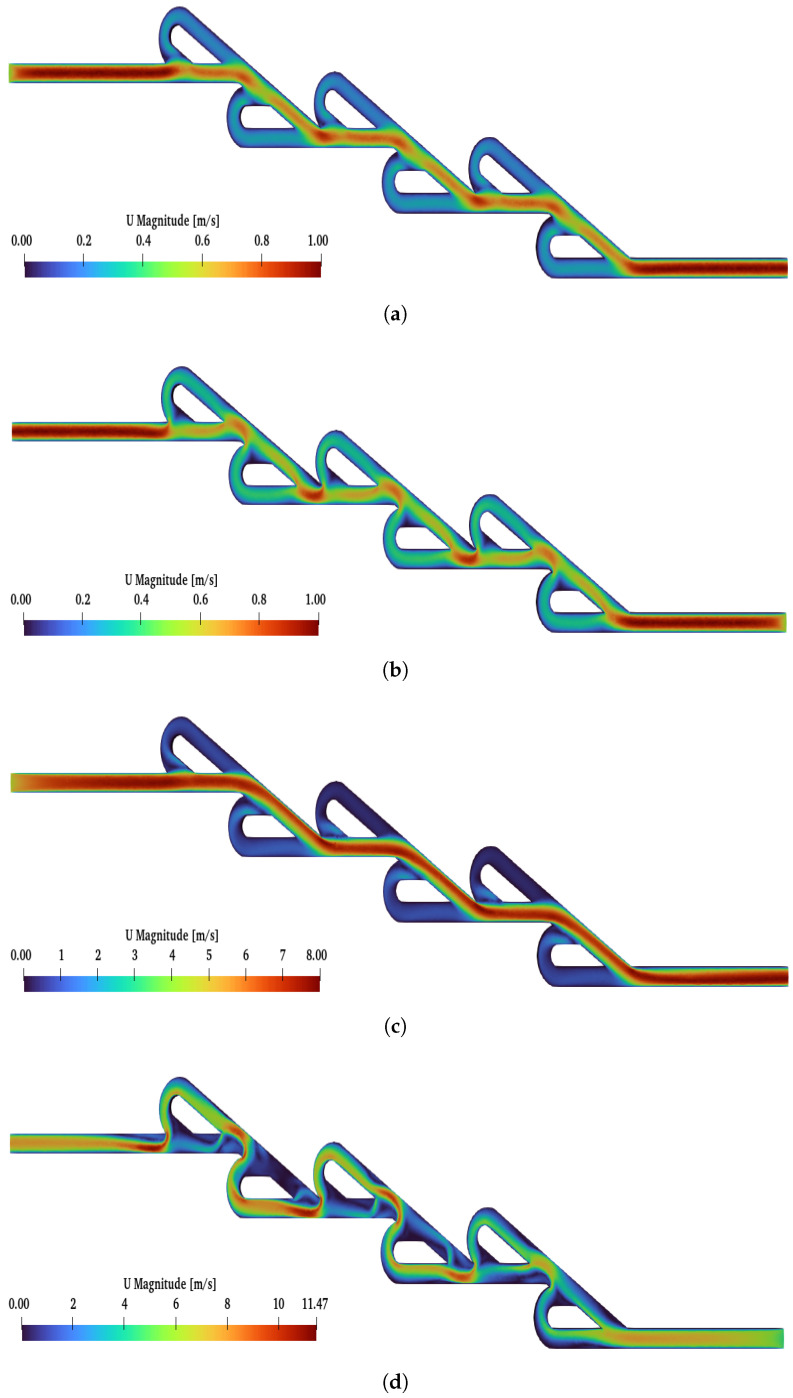
Velocity field of six-staged Tesla under (**a**) Re = 50 and forward flow, (**b**) Re = 50 and reverse flow, (**c**) Re = 450 and forward flow, (**d**) Re = 450 and reverse flow.

The post-process analysis (at inlet–outlet patches) for the six-staged micro Tesla is presented in Figure 9 with a blue line. When Re increases, this causes an increase in diodicity for the six-staged valve, as observed for the double valve. The maximum diodicity was achieved for Re=450 and was equal to D=2.76, while the minimum was D=1.03 for Re=50. In Figure 11, the flows for the two cases (Re=450, Re=50) are presented. When the forward flow is applied for both Re numbers, the maximum velocity is located at the main path, as expected. Moreover, when the reverse flow is applied for both Re numbers, the velocity is increased at the loops compared to the forward flow. Additionally, for Re=50, the magnitude of the velocity is almost uniform at the loops and has the same pattern for all loops. On the other hand, for Re=450, at the main path, many locations are observed with the minimum velocity magnitude; moreover, at the loops, the velocity is not uniform as in the case when Re=50 is applied. A significant observation is that, under Re=450 and after the second loop, there was a higher velocity at the loops. Finally, for Re=50, the maximum velocity in the valve is similar for both flows, while, for Re=450, the maximum velocity is observed for the reverse flow.

**Figure 12:**
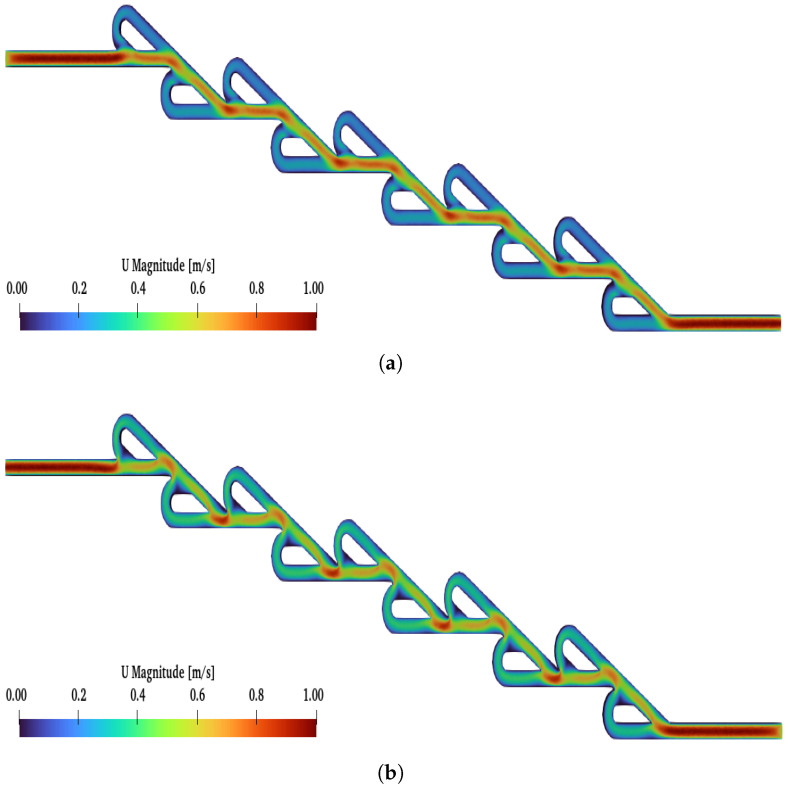

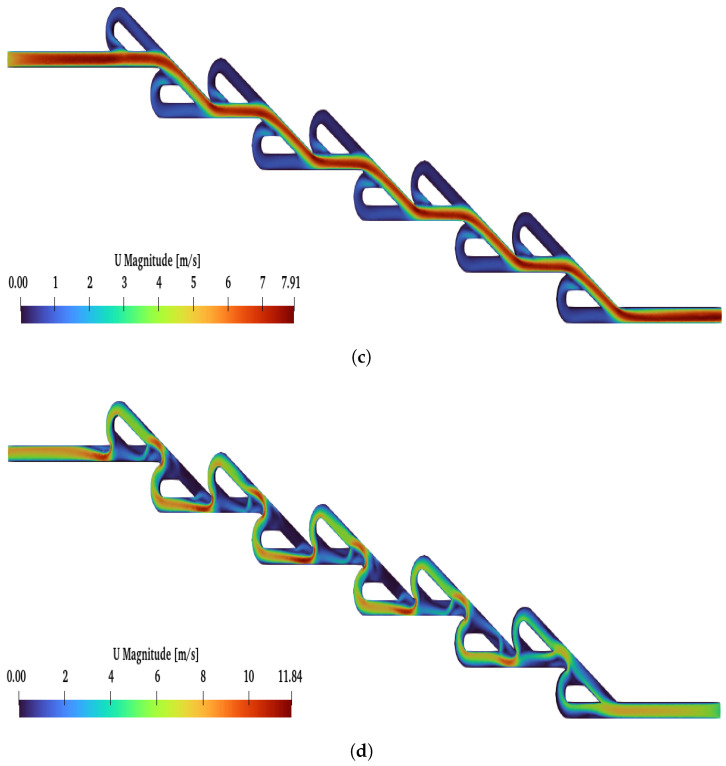
(**a**) Velocity field for ten-staged Tesla under Re = 50 and forward flow. (**b**) Velocity field under Re = 50 and reverse flow. (**c**) Velocity field under Re = 450 and forward flow. (**d**) Velocity field under Re = 450 and reverse flow.

Moreover, when the number of valves was increased further, reaching up to the ten-staged valve, the increase in Re affected the diodicity, as in the previous cases. In Figure 12, the flows for lower and higher Re numbers are presented. Additionally, in Figure 9, with the green line, we demonstrate how the increase in Re affects the diodicity. The maximum diodicity achieved was for Re=450 and was equal to D=3.58, while the minimum was D=1.04 for Re=50. The ten-staged valve’s results followed the same pattern as for the six-staged valve.

In Figure 9, the diodicity differences between these cases are also presented. Comparing the double- (N=2) and six-staged (N=6) valves, the minimum diodicity difference (ΔD) occurred at Re=50, while the maximum Δ was found equal to D=1.33 under Re=450. Moreover, as Re increased, ΔD increased also. Comparing the six-staged (N=6) and ten-staged (N=10) values in terms of the increases in Re, the ΔD increased also, while, when the number of valves increased by ΔN=4 (as before from N=2 to N=6), the maximum Δ was found equal to D=0.82. Hence, the increase in ΔD is not proportional to ΔN the number of valves. While the diodicity and Re seems to act almost linearly, the diodicity and N are not proportional, since the diodicity did not increase with the number of valves.

**Figure 13:**
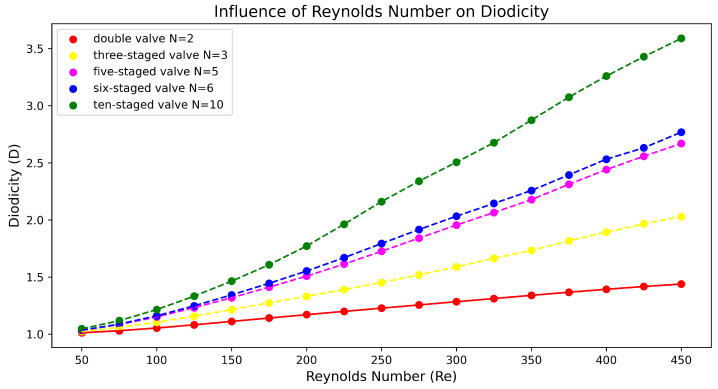
Comparison of how Re influences diodicity for various N-staged micro Tesla valves.

Finally, further simulations were performed for an even number of valves (N=3,5). The selection of the specific staged valves was based on halving N=6,10, and we verified that the diodicity was not proportional to the number of valves. The procedure followed was identical to that for the odd numbers of valves (N=2,6,10). In Figure 13, the diodicity for all N-staged valves is presented; again, as Re increases, *D* increases also. Moreover, it is now more obvious that the increase in *D* is not proportional to the increase in *N*, as minor differences appear between the five- and six-staged valves, which are clear from the comparison between N=3, N=6 and N=5, N=10, where D is not proportional to the increase in N. Moreover, in Table A2, we present the results for the simulations at the inlet–outlet patches, while, in Table A3, we present the percentage increase in diodicity between the inlet–outlet and 0.6 mm cases for different Tesla valve stages.

## 4. Discussion

From the current simulations, the direct findings are the maximum and minimum *D* for each geometry. Specifically, the maximum diodicity achieved for ten-, six-, five-, three-staged and double valves is $D_{10max}=3.58$, $D_{6max}=2.76$, $D_{5max}=2.66$, $D_{3max}=2.02$ and $D_{2max}=1.43$ under Re=450, respectively. The corresponding minimum diodicity values $D_{10min}=1.04$, $D_{6min}=1.03$, $D_{5min}=1.04$, $D_{3min}=1.02$ and $D_{2min}=1.01$ are obtained for Re=50 under ten-, six-, five-, three-staged and double valves, respectively. Even at the lowest Reynolds number examined, measurable differences in diodicity persisted among the various valve configurations, indicating that the geometric staging influences the performance even within a viscous-dominated regime. To better understand the effects of adding additional stages, a quantitative assessment of diminishing returns was performed by evaluating the incremental diodicity gain per stage (ΔD/ΔN) at Re=450; the diodicity increase per added stage follows in Table 3.

This progression demonstrates a clear reduction in diodicity enhancement per additional stage, indicating that, beyond a certain geometric complexity, the flow field saturates and additional loops contribute proportionally less to momentum redirection. In practical terms, this means that designers should avoid excessive staging, as the performance gain per added loop decreases rapidly while the fabrication cost and pressure losses continue to rise. At low Reynolds numbers (Re = 50), the incremental improvements are almost negligible, consistent with the dominance of viscous forces that suppress inertial flow separation mechanisms. For intermediate Reynolds numbers, all corresponding diodicity values across the valve configurations can be extracted from Table A2. Generally, the trend of D exhibits an increase as Re increases; the investigation of higher Re values indicates that they may not be beneficial, as, in microfluidic systems, higher flow rates may lack practical applicability. Even for Re≥250, it is difficult to find practical applications [24,25,26]. Furthermore, according to relevant studies [19], the diodicity decreases for even higher Re≥1000.

**Figure A2:**
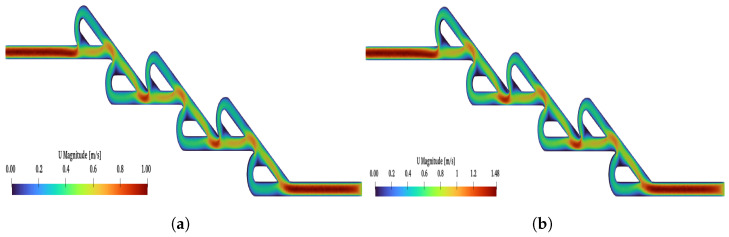

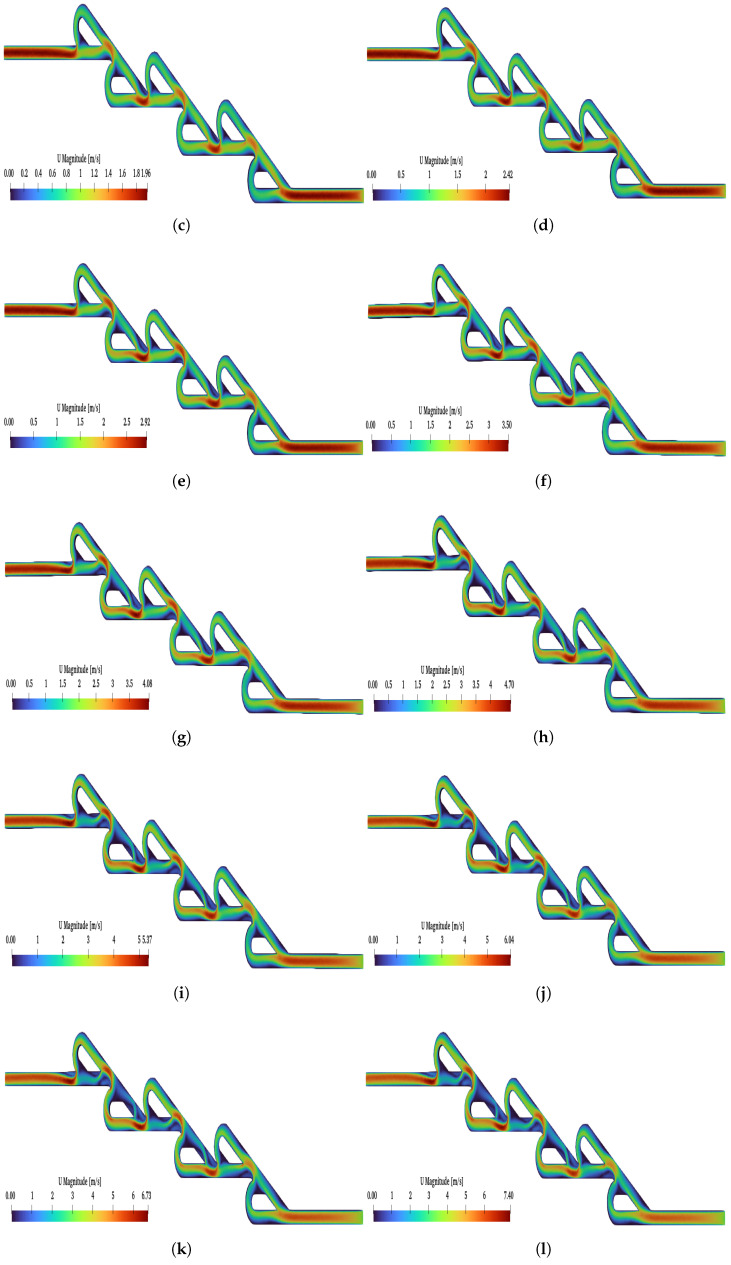

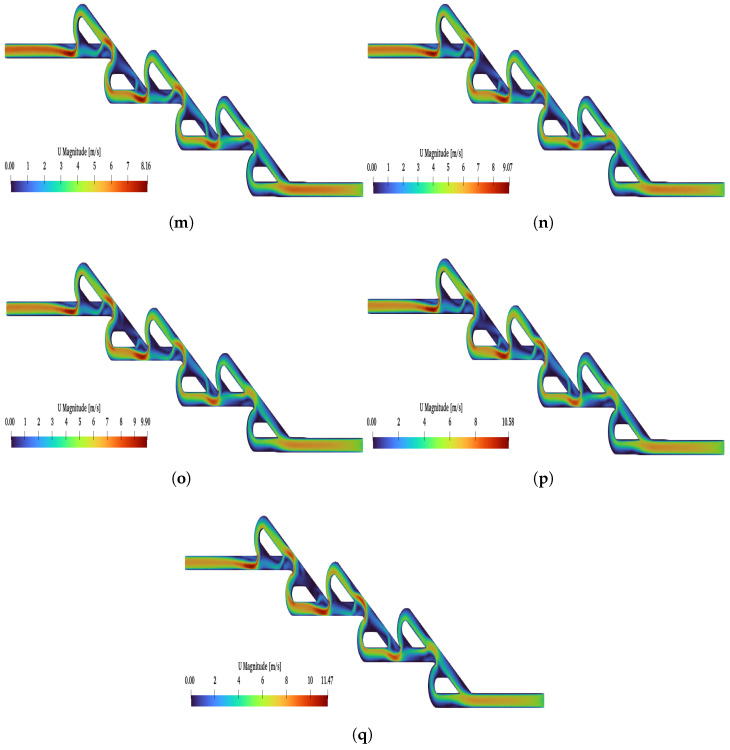
Velocity field for six-staged Tesla when reverse flow is applied: (**a**) Re = 50, (**b**) Re = 75, (**c**) Re = 100, (**d**) Re = 125, (**e**) Re = 150, (**f**) Re = 175, (**g**) Re = 200, (**h**) Re = 225, (**i**) Re = 250, (**j**) Re = 275, (**k**) Re = 300, (**l**) Re = 325, (**m**) Re = 350, (**n**) Re = 375, (**o**) Re = 400, (**p**) Re = 425, (**q**) Re = 450.

**Figure A3:**
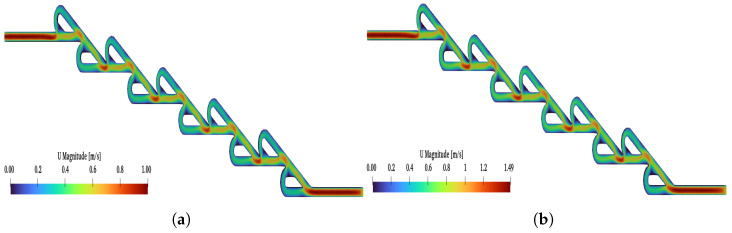

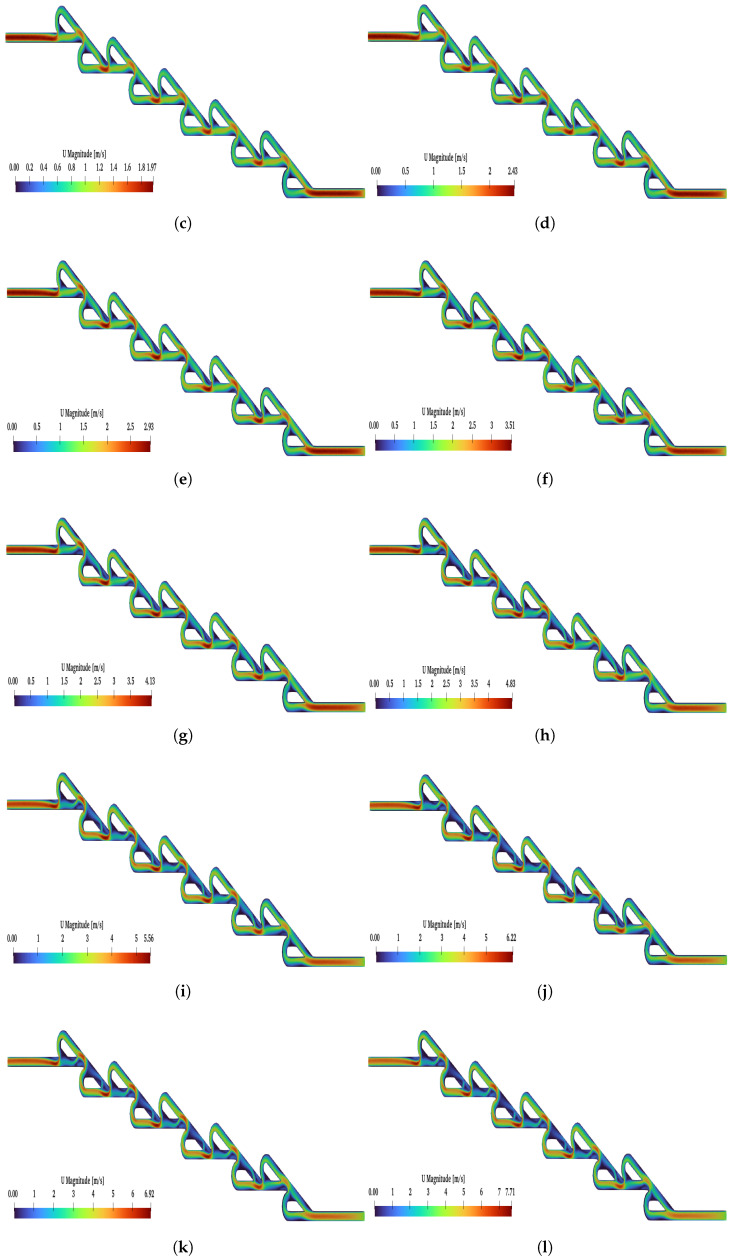

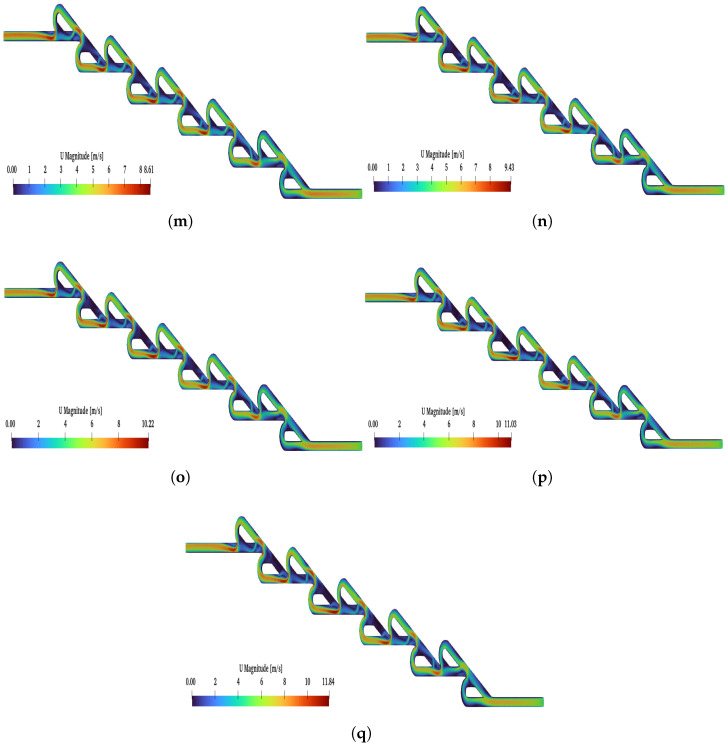
Velocity field for ten-staged Tesla when reverse flow is applied: (**a**) Re = 50, (**b**) Re = 75, (**c**) Re = 100, (**d**) Re = 125, (**e**) Re = 150, (**f**) Re = 175, (**g**) Re = 200, (**h**) Re = 225, (**i**) Re = 250, (**j**) Re = 275, (**k**) Re = 300, (**l**) Re = 325, (**m**) Re = 350, (**n**) Re = 375, (**o**) Re = 400, (**p**) Re = 425, (**q**) Re = 450.

Additionally, for the ten- and six-staged valves, when a reverse flow is applied, a critical point is observed $Re_{critical}$. Above $Re_{critical}$, the magnitude of the velocity is higher at the loops than the main path. In Figure A2 and Figure A3, the reverse flow is presented under all selected Re numbers for the six- and ten-staged valves, respectively. For the six-staged valve, the critical point is observed for $Re_{critical}\geq 150$, while, for the ten-staged valve, the critical point is observed for a lower Re ($Re_{critical}\geq 125$). Moreover, for N=6 and N=10 and a reverse flow, as the Re increased, the magnitude of the velocity decreased at the main path, but, for N=10, this phenomenon was more intense.

The most crucial finding is that, when a reverse flow is applied, regardless of Re (for Re higher than $Re_{critical}$) and N, only after the second valve does the velocity become the maximum in the loops and minimum at the main path. This is an indicator that the minimum valve number that should be used is more than two.

A direct comparison with previous computational studies reveals important insights into the necessity of three-dimensional simulations for Tesla valve analysis. Mohammadzadeh et al. [19] performed 2D simulations for Re ranging from 25 to 300 with N = 1, 3, 5, 7 and 10 stages, reporting the maximum diodicity of approximately 2.2 for N = 10 at Re = 300. In contrast, our 3D simulations yielded D=2.505 for the same configuration (N = 10, Re = 300), representing approximately 13.6% higher diodicity. This discrepancy arises from three-dimensional flow phenomena that 2D simulations cannot capture: (i) secondary flow structures in the square cross-section, (ii) three-dimensional vortex formation and interaction within the loops and (iii) cross-sectional velocity gradients that significantly affect pressure drop calculations at higher Reynolds numbers.

According to relevant research [18], the selection of Re is very important since, for Re≥300, transitional flows may occur. Thus, the simulations included Re from 50 to 200 and N=1, N=2, N=4, N=6, N=8 and N=10. In all cases, the diodicity was higher than in the present study, since Thomson achieved *D* up to 1.9 for Re=200 and N=10. Additionally, it is confirmed in the literature that the diodicity is not proportional to the number of valves, and, as Re increases, the ΔD between N=2, N=6 and N=10 also increases [18]. Moreover, as in the present study, the number of valves does not significantly affect *D* for low Re [18].

Other relevant studies [16] have investigated Re up to 1000. This study achieved maximum diodicity close to three (3) at Re=900. For Re=300, the maximum diodicity dropped to close to 1.8, which is significantly lower compared to the ten-staged valve (D=2.50), while it is comparable to the six-staged case (D=2.03) under the same Re. Meanwhile, under Re=500, *D* was less than 2.4, which is less than in the present study under Re=450.

Another relevant study that performed simulations but also experiments for N = 2, N = 4 and N = 6 and for Re within the range 50–300 achieved slightly higher diodicity compared to the current results [27]. Additionally, for almost all cases, the experiments achieved higher performance than the simulations.

Finally, it is very difficult to directly compare the values of D with the results obtained in relevant research, since the number of valves and also Re may differ. Although the performance achieved in this study is encouraging, the geometry has not yet been optimized.

## 5. Conclusions

The purpose of this work was to study the performance of double-, six- and ten-staged micro Tesla valves at various Re. Several findings arise, such as the identification of $Re_{critical}$ and the fact that D is not proportional to N. Under Re higher than $Re_{critical}$, the magnitude of velocity is higher at the loops and lower at the main path after the second Tesla (for the reverse flow) and varies with the number of valves. More specifically, for six-staged valves, it is $Re_{critical}\geq 150$, while, for ten-staged valves, it is $Re_{critical}\geq 125$ (under the simulated Re increments of this study). This discovery provides designers with specific Re thresholds for optimal performance and explains why diodicity enhancement accelerates beyond certain Re values. Notably, this 3D flow redistribution phenomenon in the reverse flow direction was not observed for the two-stage valve at any Reynolds number, indicating that such a simple geometry cannot generate sufficient inertial deflection to reorganize the backward flow momentum. This phenomenon was not reported in previous 2D studies because it requires capturing three-dimensional vortex structures and their evolution through multiple stages.

Also crucial is the understanding of the dependency of the diodicity on Re. For a low Re (≤75), the stage number has a minimal impact on the diodicity, with all configurations near D≈1. At a higher Re (≥300), N has a greater impact on D than before. This insight could be useful in optimizing the flow in various applications where flow control is demanded. These findings also provide microfluidic device designers with quantitative relationships between the Reynolds number, stage configuration and diodicity performance.

Future work will be divided into two main directions: the microscale and the macroscale. At the microscale, further investigation of intermediate Reynolds numbers (transition regime), additional staging configurations and systematic geometry optimization will be pursued. At the macroscale, efforts will focus on scale-up analysis and experimental validation to benchmark numerical predictions. Moreover, incorporating non-Newtonian fluids or pulsatile inflow conditions could further broaden the applicability of the micro Tesla valve, particularly in domains such as naval architecture (e.g., cavitation control, flow rectification in low-Re channels) and bio-microfluidic systems, where unsteady or rheologically complex flows are prevalent. Finally, coupling the micro Tesla valve design with microfabrication constraints and exploring manufacturability guidelines will help to bridge the gap between simulation outcomes and real-world implementation.

## Figures and Tables

**Table 1:** Mesh independence study.

Mesh Elements	Number of Tesla Valves	Diodicity
75,036	double	1.459
150,147	double	1.438
300,840	double	1.414
191,619	six-staged	2.731
386,381	six-staged	2.767
764,740	six-staged	2.762
306,541	ten-staged	3.427
615,535	ten-staged	3.552
1,224,889	ten-staged	3.498

**Table 2:** Simulation parameters.

Inlet and outlet dimensions (m)	$H=W=10^{-4}$	
Kinematic viscosity (m^2^/s)	$\nu =10^{-6}$	
Density (kg/m^3^)	$\rho =10^{3}$	
Dynamic viscosity (kg/m · s)	$\mu =10^{-3}$	
Boundary Conditions	Velocity (m/s)	Pressure (Pa)
Inlet	0.5–4.5 (increments of 0.25)	zero gradient
Outlet	zero gradient	0
Walls	0	zero gradient

**Table 3:** ΔD/ΔN quantification for Re=450.

Stage (N)	ΔD/ΔN
2 → 3	0.59
2 → 5	0.41
2 → 6	0.33
2 → 10	0.27
3 → 5	0.33
3 → 6	0.25
5 → 6	0.10
5 → 10	0.18
6 → 10	0.21

**Figure A1:**
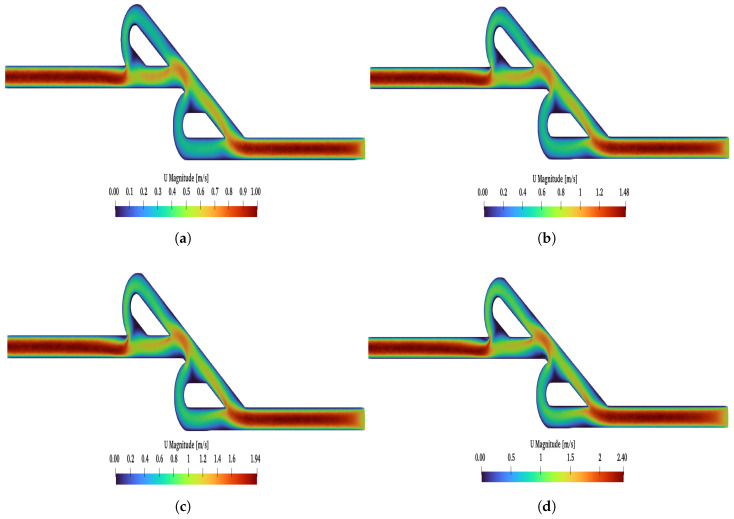

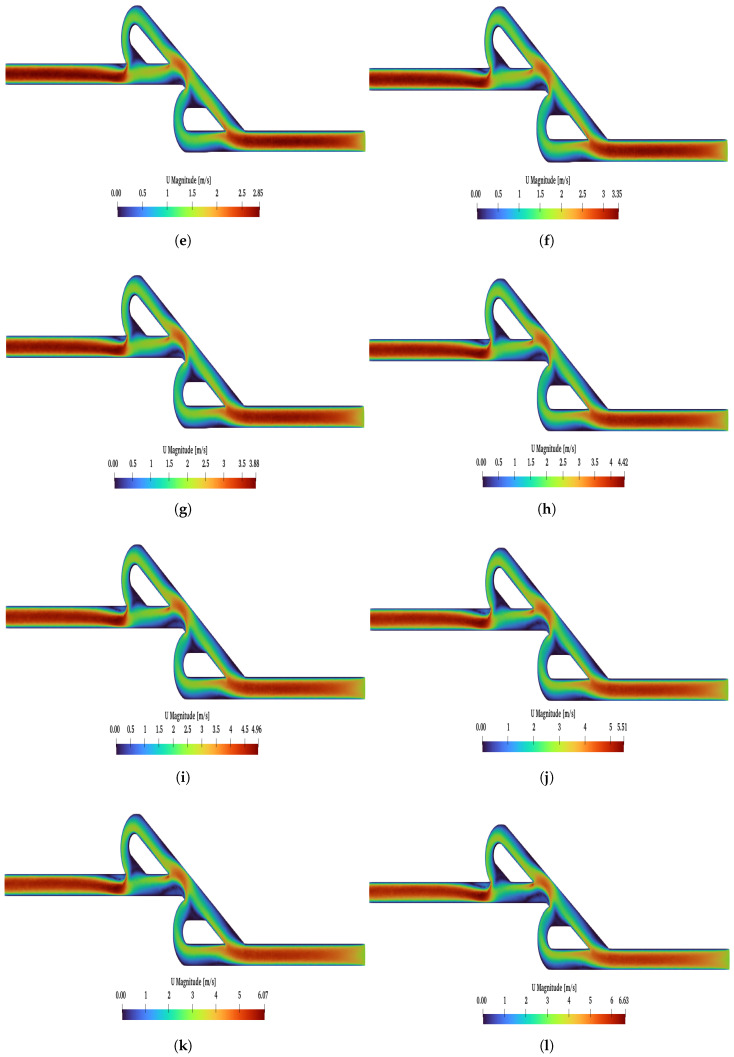

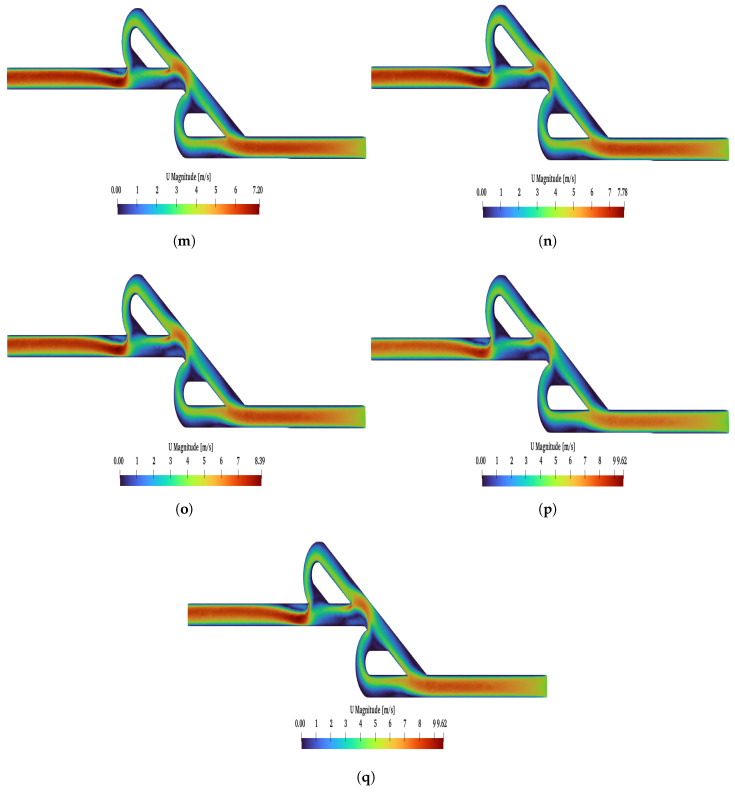
Velocity field for double Tesla when reverse flow is applied: (**a**) Re = 50, (**b**) Re = 75, (**c**) Re = 100, (**d**) Re = 125, (**e**) Re = 150, (**f**) Re = 175, (**g**) Re = 200, (**h**) Re = 225, (**i**) Re = 250, (**j**) Re = 275, (**k**) Re = 300, (**l**) Re = 325, (**m**) Re = 350, (**n**) Re = 375, (**o**) Re = 400, (**p**) Re = 425, (**q**) Re = 450.

## Data Availability

The original contributions presented in the study are included in the article; further inquiries can be directed to the corresponding author.

## Abbreviations

The following abbreviations are used in this manuscript:

D	Diodicity
N	Number of Tesla valves
CFD	Computational fluid dynamics
SIMPLE	Semi-Implicit Method for Pressure-Linked Equations
Re	Reynolds number
W	Width of micromixer
H	Height of micromixer
$L_{1}/L_{2}$	Length ratio of micromixer
*u*	Velocity of water
*p*	Pressure
ν	Kinematic viscosity
μ	Dynamic viscosity
$D_{H}$	Hydraulic diameter
*a*	Size of square inlet and outlet of micromixer
ρ	Density
Q	Volumetric flow rate
A	Surface of inlet
CGI	Convergence grid index
$\Delta P_{rev}$	Pressure difference for reverse flow
$\Delta P_{for}$	Pressure difference for forward flow
$\Delta P_{er,forward}$	Pressure difference forward relative error
$\Delta P_{er,reverse}$	Pressure difference reverse relative error
$D_{er}$	Diodicity relative error
ΔD	Diodicity difference
ΔN	Number of valves (stages) difference
$Re_{critical}$	Critical Reynolds number

## Appendix A

The inlet velocity, Reynolds number and volumetric flow rate are presented in Table A1 for all simulations.

**Table A1:** Calculation of Re number and Q.

Inlet Velocity (m/s)	Re	Q (μL/s)
0.50	50	5.0
0.75	75	7.5
1.00	100	10.0
1.25	125	12.5
1.50	150	15.0
1.75	175	17.5
2.00	200	20.0
2.25	225	22.5
2.50	250	25.0
2.75	275	27.5
3.00	300	30.0
3.25	325	32.5
3.50	350	35.0
3.75	375	37.5
4.00	400	40.0
4.25	425	42.5
4.50	450	45.0

**Table A2:** Presentation of D for each Re and valve configuration.

Re	Double	Three-Staged	Five-Staged	Six-Staged	Ten-Staged
50	1.012	1.023	1.033	1.034	1.046
75	1.029	1.058	1.083	1.087	1.117
100	1.053	1.103	1.150	1.160	1.215
125	1.081	1.158	1.231	1.247	1.332
150	1.111	1.215	1.318	1.343	1.464
175	1.141	1.273	1.411	1.445	1.607
200	1.170	1.331	1.508	1.552	1.77
225	1.199	1.390	1.613	1.669	1.962
250	1.227	1.452	1.725	1.793	2.160
275	1.255	1.518	1.841	1.915	2.339
300	1.283	1.588	1.955	2.032	2.505
325	1.311	1.663	2.065	2.144	2.676
350	1.339	1.732	2.177	2.257	2.873
375	1.366	1.817	2.312	2.394	3.074
400	1.392	1.892	2.440	2.531	3.259
425	1.417	1.966	2.557	2.631	3.429
450	1.438	2.029	2.668	2.767	3.589

**Table A3:** Percentage increase in diodicity between inlet–outlet and 0.6 cases for different Tesla valve stages.

Re	Double (%)	Three-Staged (%)	Five-Staged (%)	Six-Staged (%)	Ten-Staged (%)
50	0.451	1.062	1.327	1.307	1.154
75	1.622	2.810	3.200	3.053	2.701
100	3.211	5.042	5.505	5.216	4.545
125	5.044	7.509	7.958	7.518	6.434
150	6.993	9.996	10.341	9.758	8.207
175	8.957	12.401	12.563	11.837	9.810
200	10.889	14.673	14.687	13.787	11.339
225	12.695	16.798	16.623	15.603	12.761
250	14.396	18.865	18.393	17.162	13.921
275	16.005	20.847	19.882	18.472	14.773
300	17.435	22.635	21.176	19.670	15.366
325	18.727	24.370	22.239	20.454	15.981
350	19.913	25.809	23.323	21.312	16.514
375	21.009	27.595	24.496	22.292	17.019
400	22.006	28.727	25.520	23.156	17.409
425	23.078	30.328	26.347	23.759	17.713
450	24.098	31.699	27.086	24.509	17.699

## Appendix B

Figure A1, Figure A2 and Figure A3 present the reverse flow for double (Figure A1), six-staged (Figure A2) and ten-staged (Figure A3) micro Tesla valves for all selected Reynolds numbers.
